# Emotion in Dyads Across Adulthood

**DOI:** 10.1093/geroni/igaf122.2101

**Published:** 2025-12-31

**Authors:** Claudia Haase, Tabea Meier, Tammy English

## Abstract

Emotions in close relationships play an important role in well-being, health, and longevity across adulthood. While research has uncovered important insights into how individuals experience and express emotions in close relationships, emotional processes within dyads have received less attention. This symposium brings together four contributions that provide novel insights into antecedents and consequences of emotional functioning in dyads across adulthood. Contributions examine multiple emotion response systems (i.e., subjective emotional experiences, behaviors, physiological functioning, language), multiple emotion contexts (i.e., positive and negative), diverse dyads (i.e., healthy married couples, dementia care dyads), and different study designs (i.e., laboratory-based observations of dyadic interactions, ambulatory assessments, longitudinal studies). Dr. Meier and colleagues will present findings from a laboratory-based study on marital interaction, showing how socioeconomic status as an important macro-level factor shapes physiological linkage in middle-aged married couples. Fu and colleagues will present findings showing that language that conveys validation or invalidation during dyadic interactions can predict spouses’ emotional experiences, drawing from a laboratory-based study of married couples. Dr. Yoneda and colleagues will present findings from a large dyadic ambulatory assessment study on positivity resonance (i.e., co-experienced positive emotions) and links with momentary cortisol in spouses’ daily lives, highlighting important benefits of positivity resonance at a biomarker level in older couples. Dr. Chen and colleagues will combine data from two studies on dementia care dyads, revealing important links between physiological linkage and caregiver mental health and well-being across both laboratory and real-life contexts. Dr. English will be the discussant.

